# National audit of seven-day working care in radiology

**DOI:** 10.1259/bjro.20200046

**Published:** 2021-07-05

**Authors:** HS Bailey, P Mehrotra, KJ Drinkwater, DC Howlett

**Affiliations:** 1 Department of Radiology, Aberdeen Royal Infirmary, Aberdeen, UK; 2 Department of Radiology, City Hospitals Sunderland NHS Foundation Trust, Sunderland, UK; 3 Royal College of Radiologists, London, UK; 4 Department of Radiology, Eastbourne District General Hospital, Eastbourne, UK

## Abstract

**Objectives:**

To evaluate the extent to which our current provision of diagnostic and interventional radiology services matches existing clinical demand and future government proposals as set out in the Royal College of Radiologists published guidance on providing seven-day acute care.

**Methods:**

In June 2018, all UK radiology department audit leads were sent a questionnaire designed to assess compliance for each standard of the Royal College of Radiologists published guidance on providing seven-day acute care.

**Results:**

135 hospitals (68%) responded. Of those that responded, 96% of departments have a diagnostic radiologist rota for clinicians to discuss acute cases and review imaging and 48% of departments do not have a fully staffed consultant rota 24 h a day, seven days a week for interventional radiology. There is significant variance in MRI radiographer availability within departments, ranging from 18.8% during Saturday/Sunday evening/overnight up to a maximum of 63.9% during Saturday daytime. 11% of departments participate in a regional out of hours cross-organisation reporting rota. 40% of departments have no 24/7 RIS technical support and 34% have no PACS technical support out of hours.

**Conclusion:**

There is a wide variation in practice across radiology departments in the UK. Although there are some standards that the majority of hospitals are achieving, there is a significant short-fall in fundamental aspects of providing acute seven-day care. The multifactorial nature in which these problems have arisen means there is no easy solution to combat these issues. There is a requirement for significant investment and political commitment to improve staffing and infrastructure in order to address the current situation.

**Advances in knowledge:**

A UK wide evaluation of the current provision of seven-day working in radiology showing 54% of hospitals do not have a UK working-time regulations compliant Interventional radiology rota, severe lack of availability of acute MRI out of hours and significant deficiencies in providing technical support out of hours. A sustainable and efficient seven-day service is currently not being provided.

## Introduction

The timely availability of diagnostic and interventional radiology services is fundamental in order to optimise patient management. Modern health care is reliant on rapid access to and interpretation of these imaging examinations in order to provide high-quality, patient-centred care. The NHS Forum believes that all patients should be able to access urgent and emergency care services as well as supporting diagnostic services, delivered in a way that meets clinical standards, seven days a week.^1^ As such, there is pressure for acute hospitals to transition from the traditional five-day patient care model to a more continual seven-day service.

The Academy of Medical Royal Colleges has developed standards to deliver consistent in-patient care seven days a week. These standards include a review of hospital in-patients by an on-site hospital consultant at least once in every 24 h, seven days a week unless it has been determined that a review would not affect the patient’s management. Also, the standards recommend that consultant supervised interventions and investigations with reports should be provided seven days a week if this will change the patient’s management.^2^ The Royal College of Radiologists (RCR) standards for providing a seven-day acute care diagnostic radiology service^3^ make explicit reference to the challenges commissioners and providers may encounter to try and provide such a service. These difficulties are likely to include requiring significant levels of personnel, infrastructure, equipment and financial investment. The RCR recognises that a whole team approach is essential to ensure the delivery of an effective seven-day acute care diagnostic radiology service. It is inevitable that in order to provide such a service, additional resources will be required to fund increased staffing levels, including radiologists, radiographers, support staff, administrative and IT.

There is an ever-increasing demand on radiology services and, therefore, the workload of radiologists. The reasons for these are multifactorial. In the past five years alone, there has been a 54% increase in demand for computed tomography (CT) and a 48% increase in magnetic resonance (MR) examinations in the UK.^4^ This is partly due to an ageing population, increased multi-morbidity and the need for more complex imaging. Furthermore, the capabilities of medical imaging technology have significantly improved over the last 20 years. This has paradoxically had a compound effect on driving up the increase for radiology services, as the demand for accurate diagnostics and intervention in emergency medicine is met with the limiting factor of access to these services seven days a week. A further rise in demand is projected over the next five years. The recent 2018 clinical radiology workforce census report highlights a 9% consultant radiologist vacancy rate, therefore overcoming severe shortages alone will be particularly challenging in order to deliver an effective seven-day acute service.

This study aimed to evaluate the extent to which current provision of diagnostic and interventional radiology services in the UK matches existing and projected clinical demand and future government proposals as set out in the Royal College of Radiologists published guidance on providing seven-day acute care.

## Methods and materials

In June 2018, all UK radiology departmental audit leads listed on the RCR audit lead database were invited by e-mail to participate in the audit via a SurveyMonkey weblink to an electronic questionnaire. Institutional review board approval for this type of study is not required in the UK, as there is no individual patient data acquisition. The questionnaire comprised 58 questions (Supplementary Material 1) and was designed to assess compliance against all 14 standards (Table 1) within the published RCR document Standards for providing a seven-day acute care diagnostic radiology service.^3^ The selected targets for the standards are aspirational as they are unlikely to reflect actual practice in most centres. The RCR standards document refers to safe radiological staffing and out of hours availability with regard to diagnostic and interventional radiology services; this includes the availability of radiology support staff and staff well-being. It also compiles technical-based information on workflow efficient access to imaging, electronic patient records; voice recognition software; telephone communications and IT infrastructure including the ability to facilitate peer feedback; as well as support for radiology information systems (RIS) and picture archiving communication systems (PACS).

**Table 1.** Audit Standards and Compliance

Standard	**Indicator**	**Target**	Compliance *n/N (%)*
1. Safe radiological staffing is required to deliver satisfactory patient outcomes.	% of hospitals that have a radiologist rota for clinicians to discuss acute cases and review or report imaging out of hours % of hospitals that have a fully staffed consultant rota 24/7 for out of hours interventional radiology	100% 100%	129/134 (96) 63/122 (52)
2. Clinicians treating acutely and critically ill patients should have timely access to a radiologist when their skill is likely to aid diagnosis and/or provide therapeutic intervention.	% of hospitals that routinely provide acute care ultrasound out of hours % of hospitals that routinely provide acute care plain film ‘hot reporting’ out of hours % of hospitals that routinely provide acute care CT out of hours % of hospitals that routinely provide acute care MRI for suspected acute cord compression out of hours	100% 100% 100% 100%	119/133 (89) 41/131 (31) 132/133 (99) 98/131 (75)
3. Rostering arrangements for the delivery of acute care diagnostic radiology services should ensure adequate rest is possible before and after each shift. *Ad hoc* on-call arrangements are inappropriate. To ensure patient safety, it is important that radiologists are not fatigued and always have 11 h of continuous rest in a 24 h period, in line with the requirements of the UK Working Time Regulations.	% of hospital rota’s that routinely allow 11 h of continuous rest in a 24 h period ❏ Diagnostic Radiology Registrars ❏ Diagnostic Radiology Consultants ❏ Interventional Radiology Consultants	100%	79/82 (96) 82/124 (66) 33/71 (46)
4. Radiologists reporting from home and teleradiologists reporting outsourced imaging for acutely ill patients should have workflow efficient access to previous imaging, reports, EPRs, multiplanar processing facilities and voice recognition reporting.	% of hospitals that provide off-site radiologists routine access to multiplanar processing facilities to allow CT/MR review? ❏ Off-site Hospital ❏ Off-site Teleradiologists % of Hospitals that provide off-site radiologists with routine and consistent access to a patient’s previous radiological images on PACS ❏ Off-site Hospital ❏ Off-site Teleradiologists % of Hospitals that provide off-site radiologists with consistent access to view previous radiological reports on RIS ❏ Off-site Hospital ❏ Off-site Teleradiologists % of Hospitals that provide off-site radiologists with consistent access to consistently view the electronic patient record (EPR) ❏ Off-site Hospital ❏ Off-site Teleradiologists % of Hospitals where radiologists use voice recognition software to report ❏ On-site Hospital ❏ Off-site Hospital ❏ Off-site Teleradiologists	100% 100% 100% 100% 100%	81/102 (79) 79/80 (99) 97/111 (87) 60/96 (63) 96/109 (88) 69/95 (73) 58/101 (57) 4/83 (5) 121/130 (93) 46/107 (43) 71/75 (95)
5. Robust IT infrastructures should be in place to support image and report sharing.	% of departments that can routinely obtain regional imaging that has been performed outside their organisation out of hours?	95%	76/130 (58)
6. Radiologists reporting acute imaging should be supported by secretarial or clerical staff to facilitate the communication between radiologists and the referring doctors. This particularly applies to critical, significant or unexpected report communication.	% of hospitals where radiologists routinely have access to clerical support out of hours to facilitate communication for critical, significant or unexpected findings? ❏ On-site Hospital ❏ Off-site Hospital ❏ Off-site Teleradiologists % of hospitals that routinely have clerical support available out of hours to type up urgent reports during technical failure	95% 95%	7/129 (5) 3/110 (3) 22/84 (26) 3/130 (2)
7. There should be clarity from the provider (*i.e.* base hospital) about what acute care services are provided on site on a 24 h basis and referral protocols should be agreed.	% of departments that have clear local procedures for out of hours provision of acute care services ❏ US ❏ CT ❏ MRI Cord Compression ❏ Interventional Radiology	100%	57-108/130 (44-83) 121-125/130 (93-96) 50-101/130 (38-78) 65-70/130 (50-54)
8. RIS and PACS support should be available seven days a week.	% of hospitals that provide RIS technical support out of hours 24/7 % of hospitals that provide PACS technical support out of hours 24/7	100% 100%	78/130 (60) 86/130 (66)
9. To provide an effective acute care diagnostic radiology service, it should be delivered as part of a provider’s delivery of all seven-day acute care services and not as an isolated service.	% of departments that have a chaperone routinely available out of hours for USS or image guided procedures % of departments that have clerical support routinely available out of hours to book exams or ensure radiological exams are ready for reporting % of departments that have routine availability of a translator out of hours to assist with ultrasound scans or interventional procedures	100% 100% 100%	88/130 (68) 37/130 (28) 52/130 (40)
10. IT systems should enable efficient electronic text feedback to all radiologists involved in emergency imaging or intervention, to benefit patients and facilitate learning.	% of hospitals that have IT systems to allow electronic communication between radiologists % of hospitals that routinely feedback discrepancies to the reporting radiologist, radiographer or sonographer	100% 100%	82/130 (63) 123/130 (95)
11. All radiologists reporting imaging of acutely ill patients or intervening on them should have well-defined efficient telephone communication systems that permit urgent discussion with clinicians who have overall responsibility for such patients.	% of hospitals that provide off site radiologists with 24 h telephone communication access to the referring clinician to permit urgent discussion of an imaging report? ❏ Off-site Hospital ❏ Teleradiologist	100%	105/111 (95) 87/97 (90)
12. Health providers and commissioners that sign up to providing seven-day acute care diagnostic radiology services must ensure that such services are adequately staffed and resourced to provide a sustainable high-quality service, protect the health and well-being of staff and to ensure that patient safety is not compromised.	% of hospitals that do not have unfilled gaps on the onsite consultant on call rota	100%	129/130 (99)
13. When any aspect of acute radiology services cannot be provided on a 24 h basis, this should be formally reported and placed on the provider’s risk register. Business cases for alternatives for providing that acute radiology service should be urgently developed and discussed with the provider’s management.	% of hospitals that if an acute radiology service cannot be provided but is considered essential to the organisation, place it on the risk register	100%	103/130 (79)
14. Large networks of radiologists may facilitate sustainable acute seven-day rotas.	% of hospitals that participate in a regional out of hours cross-organisation reporting rota?	No Target	14/129 (11)

Where an institute has answered ‘Not-Applicable’ or ‘Don’t Know’, the denominator has been adjusted and is reflected in the compliance.

For Standard 7 each out of hours time frame was evaluated separately, therefore a range has been given.

As Standard 14 states that radiology networks *may* facilitate sustainable acute seven-day rotas, a target was not set.

The study focused on compliance with the audit standards out of hours; therefore, unless otherwise specified, all results and figures relate to out of hours only. In order to standardise data collection, a full set of definitions were provided (Table 2).

**Table 2.** Audit Definitions

Term	Definition
Acute Care	All critically ill in-patients/emergency departments patients that require immediate diagnostic/interventional imaging.
Non-acute Care	All in-patients and out-patients that do not fall into the acute care category
Inpatient non-acute Care	All ward admitted in-patients that do not require immediate diagnostic/ interventional imaging.
Elective Care	All out-patients
Onsite Hospital Radiologist	A radiologist primarily employed by the hospital and reporting from within the hospital.
Offsite Hospital Radiologist	A radiologist primarily employed by the hospital but reporting offsite (*e.g.,* home reporting).
Offsite non-hospital radiologist/ teleradiologist	A radiologist usually working remotely for a teleradiology company.
Normal working hours/ weekday	Monday–Friday generally 8 am–6 pm
Daytime	Generally 8 am–6 pm
Evening	Generally 6 pm–10 pm
Overnight	Generally 10 pm–8 am
Out of Hours	Monday–Friday generally 6 pm–8 am and weekends
Elective weekday evening work	Non-acute radiology performed generally sometime between 6 pm–10 pm, Monday–Friday (*i.e.,* out of normal working hours).
Elective weekend work	Non-acute radiology performed generally sometime between 8 am–6 pm on Saturday or Sunday.
Weeknight work	Monday–Friday generally between 10 pm–8 am

An e-mail reminder was sent in August 2018, and data collection ceased in December 2018. In order to evaluate potential bias between respondents and non-respondents, the home nation, that is, the responders constituent country within the UK was used. Data analysis was performed using Microsoft Office Excel 2010. “Don't know” responses were removed, and overall results adjusted accordingly.

## Results

### Characteristics of participating institutions

One hundred and thirty-five responses from the 198 radiology departments eligible to participate were received. One hospital completed the home nation section only and, therefore, was removed from the data analysis, giving an overall response rate of 68%. Responses from radiology departments across the UK were received, with representation from all four home nation countries. The proportion of respondents to nonrespondents differed in England by 0%, in Northern Ireland by 1% and in Scotland and Wales by 6% (Table 3). The majority of responses (75%) were from District General Hospitals. Eleven responses (7%) were from a mixture of adult-only, paediatric only and single speciality care centres. 93% of respondents had a fully operational emergency department, with the majority also providing: surgical assessment units; medical assessment units; paediatric medical/ surgical in-patient services and stroke thrombolysis services. A smaller proportion offered Neurosurgery and Cardiothoracic services - 16 and 22%, respectively, thus giving a representative sample of radiology departments across the UK.

**Table 3.** Proportion of respondents to non-respondents

	Respondents (*n* = 134)		Non-respondents (*n* = 63)	
	n	%	n	%
England	104	78	49	78
Scotland	19	14	5	8
Northern Ireland	6	4	3	5
Wales	5	4	6	10

### Safe radiological staffing

The diagnostic radiologist rota for clinicians to discuss acute cases and review imaging is provided in the majority of hospitals (94%) by a combination of onsite and offsite hospital Consultants, teleradiologists and radiology registrars. In a small proportion (5%), this service is partly provided by regional radiology networks. With regard to being able to provide a continuous interventional radiology rota, the departments that can facilitate this do so through a combination of hospital (56%), regional network (29%) and mixed (16%) rota contribution.

If there are gaps in the onsite consultant on-call rota, 96% of departments use internal cover by hospital radiologists and 50% of departments use locum and teleradiologist cover. If a consultant works additional time at weekends, this is always recompensed by various means including extra pay at a sessional rate/ locum rate; time in lieu or job planned accordingly.

**Figure 1.**
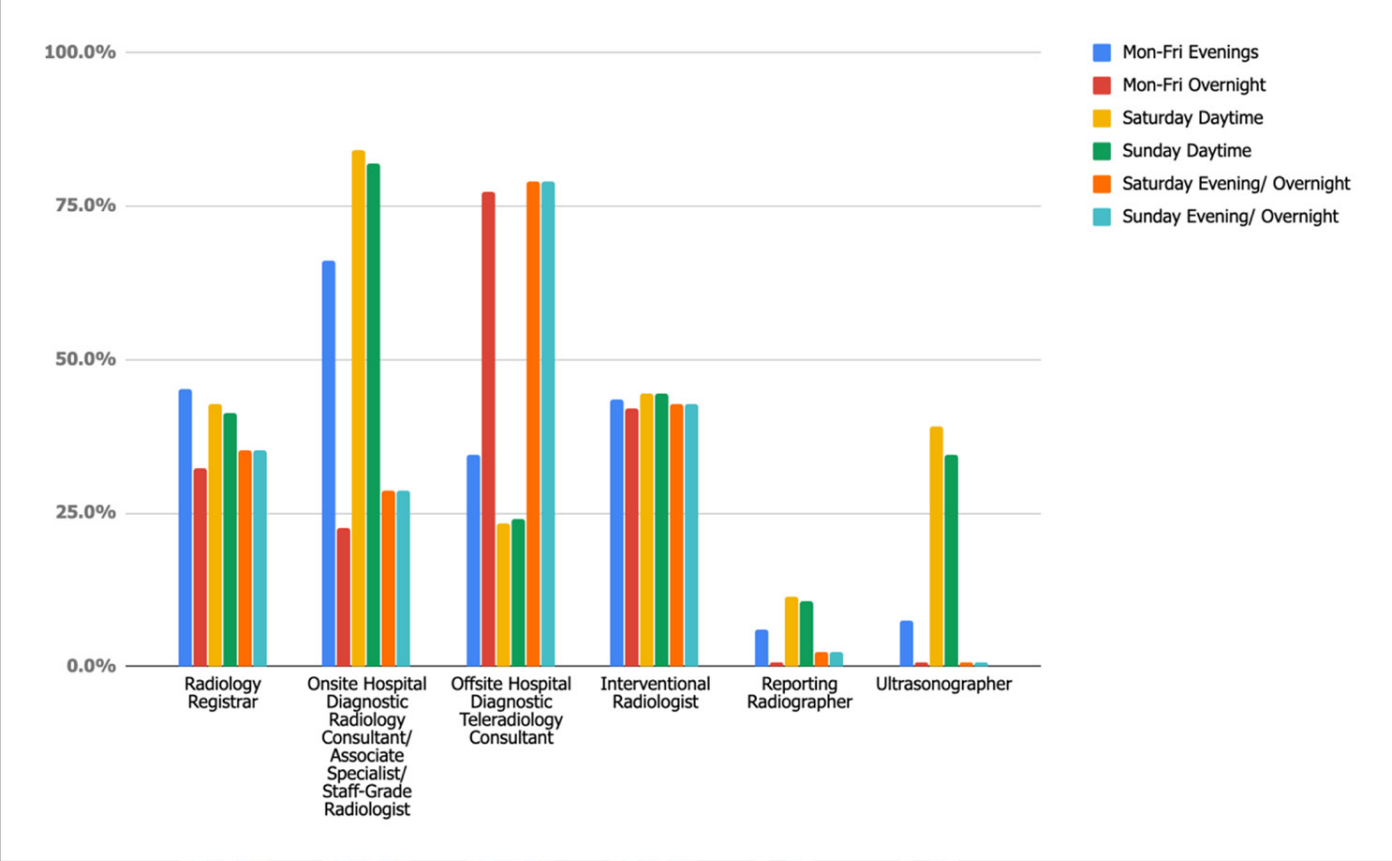
Standard 1: Radiology Departmental Reporting Staff Availability.

Figure 1 depicts the availability of departmental radiology staff out of hours. When diagnostic radiology reporting staff availability is concentrated to diagnostic consultants alone; availability within departments ranges from 88.7% Monday–Friday Overnight to a maximum of 97.7% Saturday and Sunday Daytime (audit standard 100%). In those hospitals where there was no routine diagnostic consultant availability, this was partially addressed by registrar availability, but there was no additional provision from reporting radiographers. Despite the additional coverage provided by registrars which increased compliance to 95.5% Monday–Friday Overnight, up to a maximum of 98.5% Saturday and Sunday Daytime, the audit standard of 100% was not achieved.

Radiographer availability varies significantly between imaging modalities, with as few as 18.8% departments having MRI radiographers available on Saturday/Sunday evening/overnight; increasing to 63.9% for Saturday daytime. There was increased availability of X-ray and CT radiographers within departments, ranging from 91% for CT during Monday–Friday overnight up to 97.7% for X-ray during Monday–Friday Evenings.

Only 41 departments (31%) offer “hot reporting” of plain films, of which the majority (78%) is provided by onsite hospital consultants (or equivalent) during Saturday daytime. For this study, a staff grade or associate specialist was deemed as “equivalent” to a hospital consultant. Both radiology registrars and reporting radiographers contributed similarly to plain film “hot reporting” during Monday–Friday evenings and weekend daytime. Limited weekday overnight “hot-reporting” cover was mainly provided in departments (12%) by radiology registrars. Weekend evening reporting overnight within departments is primarily carried out by in-house reporting staff; radiology registrars and on-site hospital Consultants (both 12%) and reporting radiographers (7%).

**Figure 2.**
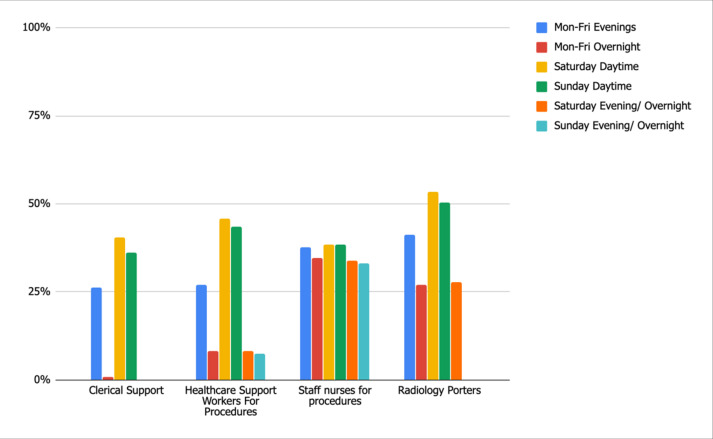
Standard 1: Radiology Support Staff Availability.

There is a significant lack of availability of radiology support. All units reported there was no clerical support availability for Saturday/Sunday evening/overnight, with only 41 and 36% availability Saturday and Sunday daytime, respectively. Similar results are seen with respect to all radiology support staff (Figure 2).

### Timely access to diagnostic and interventional radiology

The availability of all imaging modalities was evaluated for out of hours acute care and elective/outpatient or non-acute inpatient care collectively.

### Out of hours USS service

**Figure 3.**
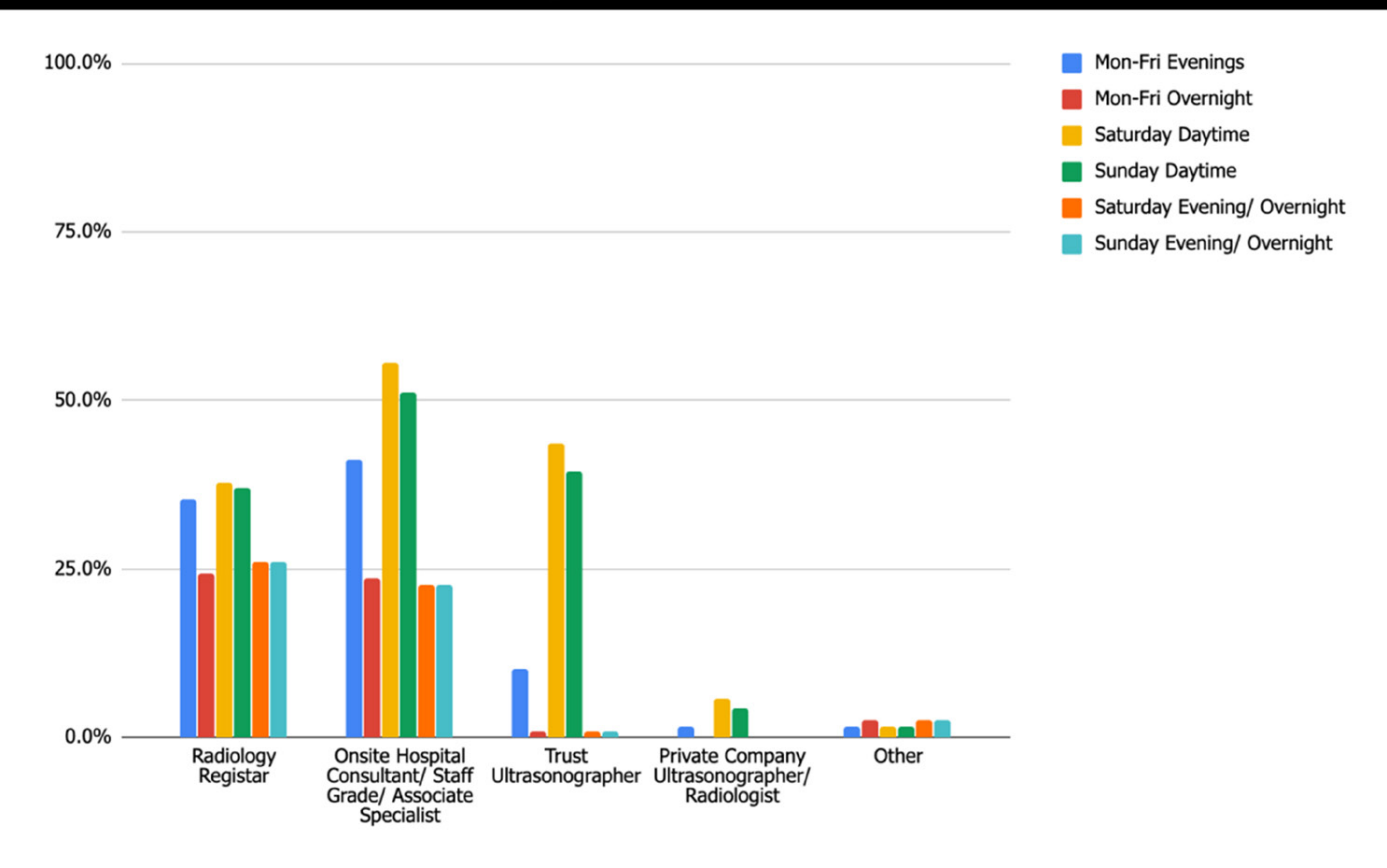
Standard 2: Acute Care Ultrasound Operators.

One hundred and nineteen (89%) departments offer an acute care ultrasound service at one or more times during the specified out of hours time frames (audit standard 100%). Monday–Friday evening and overnight scans are mostly carried out by radiology registrar and onsite hospital Consultants (or equivalent) (35.3 and 41%, respectively). There is a more significant contribution from hospital sonographers during Saturday and Sunday daytime; 43.7 and 39.5%, respectively (Figure 3). 64% of departments are also able to offer elective/outpatient or non-acute inpatient ultrasound out of hours, the vast majority of which (76%) are carried out by hospital-based sonographers during Saturday daytime. Radiology registrars and onsite hospital consultants contribute to this departmental service much less, averaging 18 and 32%, respectively. Privately provided sonographers/radiologists extend availability further, performing 21% of elective care ultrasounds on Saturday daytime.

### Out of hours CT service

**Figure 4.**
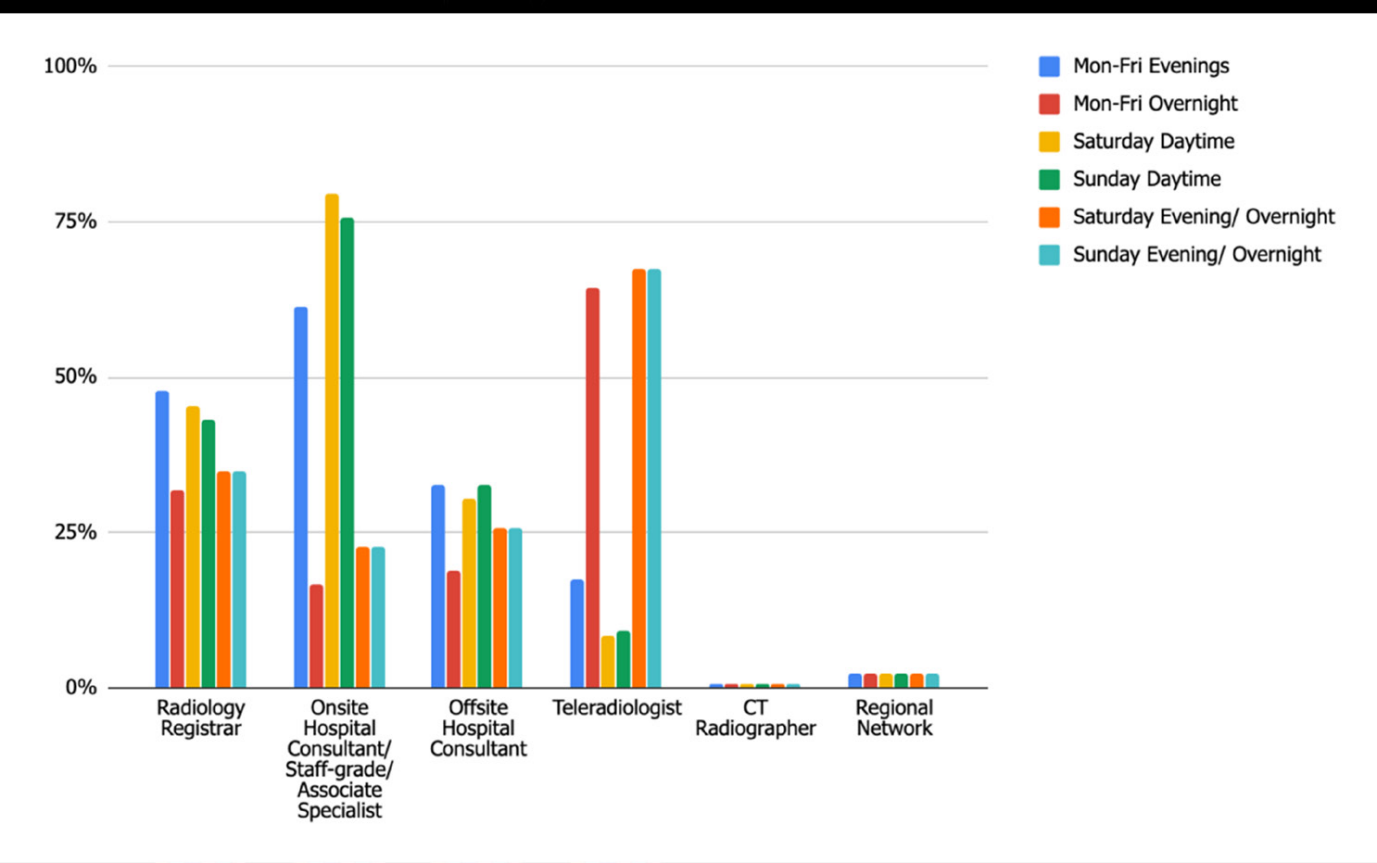
Standard 2: Acute Care CT Reporting.

One hundred and thirty-two hospitals (98%) offer an acute CT scanning service, rising to 99% during Saturday daytime (audit standard 100%). Acute care CT reporting is provided 99% of the time during one or more of the out of hours specified time frames. The majority of reporting during Monday–Friday evenings and Weekday daytime is provided by in-house reporting staff. However, teleradiologist reporting cover is substantial during both weekday overnight (64%) and weekend evening/ overnight (67%), (Figure 4).

There is a considerable amount of elective care CT provided in departments out of hours, especially Monday–Friday evenings (69%) as well as Saturday and Sunday daytime (77 and 69% respectively). As expected, very few departments provide this service overnight (average 6%). Despite the large volume of scans performed, only 43% of departments offer reporting sessions for these elective/outpatient or non-acute inpatient CT out of hours. Interestingly, the majority of the scans are reported by onsite hospital consultants (or equivalent) out of hours, mainly during weekday evenings (76%), but also Saturday and Sunday daytime (71 and 60%, respectively). In contrast, outsourced teleradiology reports only account for 10–14%.

### Out of hours MRI service

**Figure 5.**
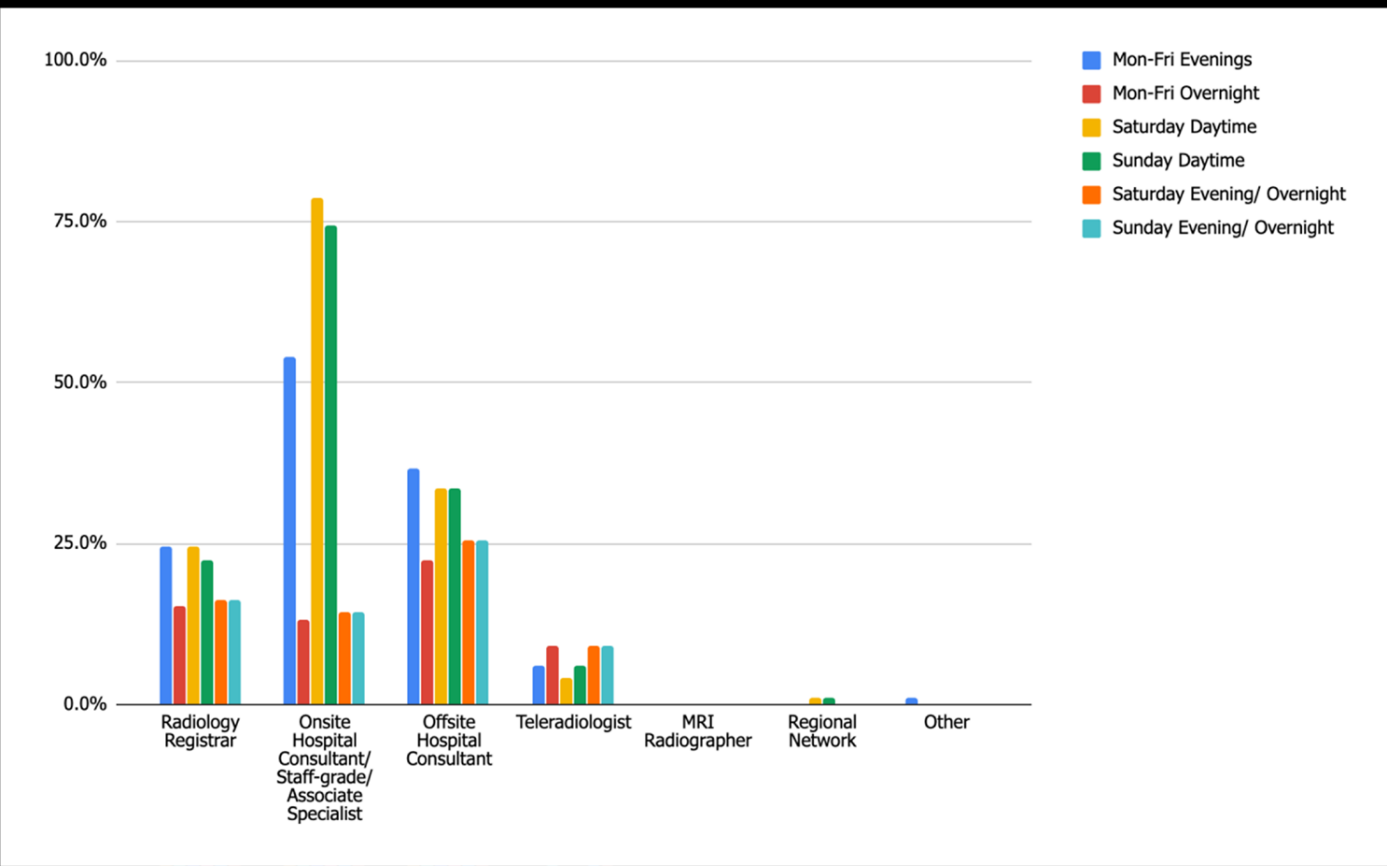
Standard 2: MRI Reporting for Suspected Acute Cord Compression.

**Figure 6.**
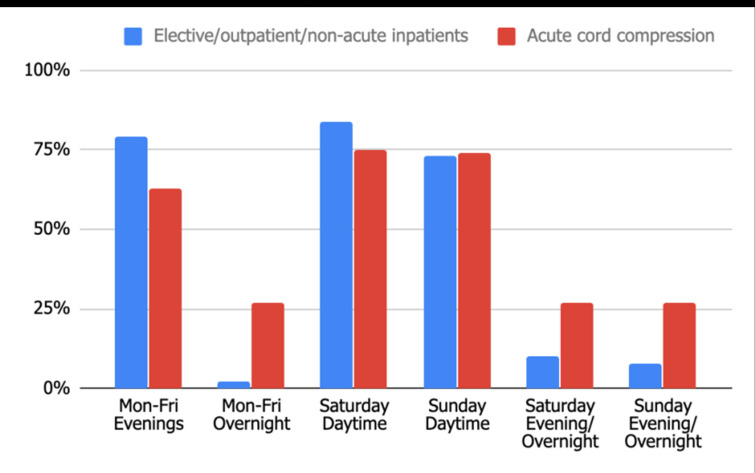
Elective *vs* Acute cord compression MRI.

Acute MRI access for suspected cord compression varies considerably between hospitals (audit standard 100%). Saturday and Sunday daytime availability is most frequently offered (75 and 74% respectively), whereas weekday and weekend evening/overnight is only available in 27% departments. Ninety-eight hospitals (75%) routinely offer suspected acute cord compression reporting. Figure 5 depicts which radiology staff contribute to this reporting commitment. There is, however, a significant elective/outpatient/non-acute inpatient MRI facility in many departments with 79% occurring on weekday evenings as well as 84 and 73%, respectively, on Saturday and Sunday daytime (Figure 6).

**Figure 7.**
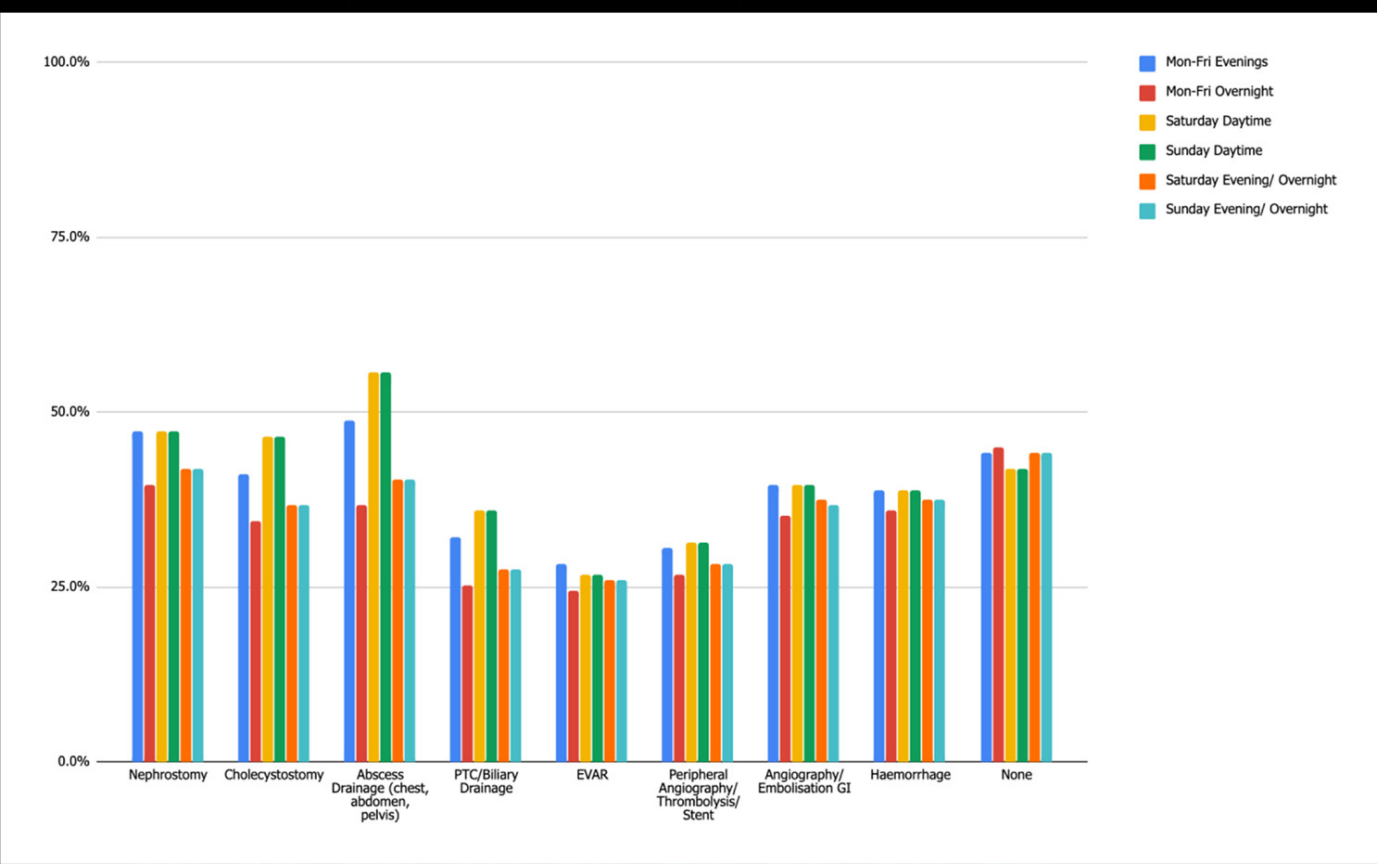
Standard 2: Acute Care Image-Guided Procedure Availability.

Figure 7 illustrates the routine availability of acute care image-guided procedures. 42–45% of departments have no facility for acute care image-guided procedures out of hours (audit standard 100%).

### Acute care service provision

The majority of departments (93–96%, depending on the specific out of hours time frame) have clear local procedures for out of hours provision of CT (audit standard 100%). However, regarding the other imaging modalities, namely, ultrasound, MRI and interventional procedures, this is considerably more varied, and as few as 38% have local procedures in place for acute MRI in cases of suspected cord compression on Sunday Evening/overnight. Furthermore, ultrasound local procedure policy availability ranged from 44% Saturday Evening/ overnight to 83% Saturday Daytime. An out of hours interventional radiology local procedures policy is only in place in 50–54% of hospitals.

## Discussion

In order to deliver satisfactory patient outcomes and high-quality patient care, safe radiological staffing is a requirement. Despite the reported workforce shortages, the majority of departments that participated in this study met the audit standard for providing a 24/7 rota for acute diagnostic care (standard 100%, achieved 96%). The responses indicate that many units have incorporated teleradiology and off-site hospital consultant reporting as an integral part of the service. It is likely that without this, a near-continuous acute diagnostic rota would not exist.

Remarkably, only 1% of gaps on the consultant on-call rota are left unfilled. However, the fact that many departments use internal cover when there is a marked shortfall of diagnostic consultant radiologists is another facet for consideration with regard to increasing workload and its consequential impact on the growing dilemma of burnout in radiologists.^5^ It is vital that in order to help address this matter, when a service cannot be provided it is placed on the risk register (audit standard 100%), yet in 21% of organisations, this is not the case. Currently, only 11% of hospitals utilise regional network reporting rotas. Many regions are actively engaged in developing such regional networks. However, there are significant IT, PACS and workforce issues to overcome before this can be achieved, necessitating high financial input. As a result, these initiatives are taking far longer than initially envisaged to embed. Given that the current rate of spending on teleradiology services in the UK has spiralled to £165 million, triple that from 2014 and equivalent to 1887 consultant radiologist salaries, if and when these networks are established, this may reduce outsourcing expenditure.

Despite the 100% fill rate for clinical radiology training posts in recent years, 71 hospitals (53%) have no registrar out of hours provision. According to the recent 2018 workforce census report, clinical radiology trainee numbers need to treble to close the forecast gap between supply and demand. This expansion may provide further out of hours roles for radiology registrars, thereby assisting emergency radiology provision. The census report also focuses on the problem of early retirement resulting in the loss of valuable expertise.^4^ A proposed action which may at least help to stem the loss of experienced consultant radiologists would include incentivising a delay in retirement. Concerns over work-life balance and existing pension tax penalties are felt to be particularly influential factors in this matter.

For patient safety, acute hospital trusts must ensure that they can provide emergency services 24/7 365 days per year and irrespective of hospital size and geography, timely access to interventional radiology should be no exception. Despite this, there is a significant shortfall in the provision of an out of hours interventional service, (audit standard 100%) with 48% of hospitals unable to uphold a continuously staffed rota. In hospitals where a 24/7 rota is upheld, it is done so with significant support (44%) from either regional networks or a mixture of both hospital rotas and regional networks. In addition to this, 54% of interventional radiology rotas are non-compliant with the UK working time regulations in allowing 11 h of continuous rest in a 24 h period,^6^ (audit standard 100%). It is perhaps not unsurprising then that a recent study^7^ has highlighted a burnout prevalence of 71.9% in interventional radiologists. The results show that this was even more marked in female interventional radiologists working more than 80 h per week.

Additionally, acute care image-guided procedures are offered in substantially fewer hospitals than the 100% audit standard. Of the procedures stated, endovascular aortic repair is by far the most infrequently offered owing to its highly specialist nature. However, even excluding complex interventional radiology procedures, image-guided abscess drainages, for instance, are still only offered in 56% of departments during Saturday and Sunday daytime. These results suggest there is a high reliance on interventionalists, even though certain procedures may not necessitate their expertise. This may be because most diagnostic radiologists do not undertake these interventions routinely and so are deskilled and unable to perform them safely. There are also the medico-legal implications of performing such procedures when they do not carry them out regularly.

With regards timely access to radiologists, diagnostic Consultant radiologist availability almost achieved the 100% audit standard during the daytime at the weekend (97%) and increased further when the availability of a registrar was also included (98.5%). These results were broadly in line with the availability of CT radiographers, varying from 95–99%. As well as access to acute care CT scans (98–99%) and acute care CT reporting (99%), in-house Consultant radiologists provide the majority of acute care CT reporting during the weekday evenings and weekend daytime with a high reliance on teleradiologists to provide this extended service overnight. These results presumably reflect the national shortage of radiologists, with insufficient numbers available to report overnight in order to have sufficient rest to report the following day.

One of the more pressing problems highlighted by this study is the reduced availability of acute MRI out of hours and limited departmental availability of both MR radiographers and reporting staff. Concerning acute MRI in suspected cord compression, the current NICE guidelines state this should be performed within 24 h.^8^ However, this is at best offered in 75% of hospitals during the day on weekends; with only 27% of departments able to provide this service overnight. The departments which can offer this MRI service overnight are a mixture of both tertiary referral centres and district general hospitals. Interestingly only 44% of the departments have neurosurgery on site. MRI reporting of suspected acute cord compression is only available in 75% of departments with the majority reported by in-house Consultant radiologists, whether that be an onsite/ offsite basis.

Of more critical concern, only 18.8% of departments have MRI radiographer availability on Saturday/Sunday evening/overnight; increasing to 63.9% for Saturday daytime. Interestingly there is more capacity for elective care MRI than there is for acute care suspected cord compression MRI in almost all out of hours time frames (Figure 6). There are likely to be various factors resulting in this. The UK workforce 2018 census report announced a 48% increase in MRIs performed over the last five years; this ultimately results in increased waiting times for non-urgent examinations if capacity is not increased. In light of the ever-increasing demand for MRIs, the current lack of alternative reporting options, as well as insufficient radiographer availability, is a highly sensitive issue which needs addressing as a matter of urgency. One possibility could be a regional MRI unit for out of hours emergencies. Although initially there would be financial implications; overall, this is likely to prove beneficial for patient care and in the long term could be cost-effective.

A similar situation is also occurring with ultrasound imaging out of hours. Sonographers in many departments are delivering a high proportion of the service for both acute and non-acute patients. Again, the elective service capacity in many departments out of hours is higher than the acute care provision. A potential option moving forward could be to utilise sonographers expertise further with regard to out of hours acute care imaging. This proposed solution is likely to increase sonographers unsociable hours commitment, which may prove challenging. However, it is clear from these results that many departments are already developing this service. In doing so, departments relieve some of the workload from radiologists who can then redirect their attention to other imaging modalities that are currently outsourced, thereby reducing some of the financial burden implicated in many trusts.

This study highlights significant deficiencies in the provision of technical support to reporting radiologists out of hours. This is evident in key areas such as obtaining external imaging and also previous imaging within the same organisation, (audit standard 100%). Furthermore, multiple departments report that even when the facility to retrieve imaging is available, it is not consistent. There are very few departments where the teleradiologists and offsite hospital radiologists have access to the patient’s electronic patient record (5 and 57% respectively), which is a substantial deviation from the 100% audit standard. All radiologists reporting the imaging of acutely ill patients must have well-defined, efficient telephone communication systems to permit urgent discussion with the responsible clinician for the patient, (audit standard 100%). Nevertheless, 5% of departments do not have this 24 h a day telephone communication for offsite hospital radiologists, and 10% of departments do not have this 24 h a day telephone communication for teleradiologists. Limiting access to relevant clinical information and hindering communication has significant implications for patient management. The recent coronavirus global pandemic has forced many radiology departments around the country to vastly improve their radiology communication systems at an accelerated rate due to indwelling inefficiencies being put under serious scrutiny. This has included increased implementation of the previously underutilised video conferencing tools. Additionally, these improvements have not only increased efficiency internally between departments and remote workers but also aided the transfer of communications between teleradiology services and hospital radiology departments.

Only 63% of departments provide electronic means of communication between radiologists (audit standard 100%). Mechanisms must be in place to assist in providing rapid peer feedback and to facilitate second opinions in a manner which is instantaneous, secure and not cumbersome. Without these means, feedback will not occur promptly and therefore is of little use. Electronic communications can also assist in capturing some of the peer reviews which take place every day in radiology departments.

The proportion of respondents to nonrespondents differed very little by geographical location. This, together with an overall response rate above 60%,^9^ is probably sufficient to assume the risk of serious response bias is negligible. Only radiology departmental audit leads listed on the RCR audit lead database were invited to participate in the survey. This database is regularly updated but its maintenance is reliant on the response of individual hospitals. Therefore, a request may not have been received or actioned by departments either if they were not on the list, or if incorrect details were held. One hospital completed the home nation section only and therefore was removed from the data analysis.

### Key suggestions

In order to address some of the shortfalls in providing a consistent and timely seven-day service as highlighted in this study, Trusts could consider development of regional services particularly for MRI and for acute image guided procedures. CPD training may enable many radiologists to retain and expand their skills in MRI interpretation and acute image guided procedures to support these services. Utilisation of sonographers expertise with greater incentivisation and engagement may assist the provision of out of hours acute care imaging. Finally, to address radiographer staffing deficiencies, internal radiographer rotations and support for radiographer training may broaden skill sets and overcome on call vacancies.’

## Conclusion

It is clear from these audit results that there is a wide variation in practice across radiology departments in the UK. Although some departments are achieving the RCR standards for providing a seven-day acute care diagnostic radiology service, at a national level, none of the standards have been met. Furthermore, there is a significant short-fall in various fundamental aspects of providing acute seven-day care, which comes at a high cost to patients. It is hard to escape from the fact that to provide an effective and genuine seven-day service, it is going to be complex, expensive and take time. Collaboration between governmental agencies and professional bodies is of paramount importance to fund investment for future radiology trainees, radiographers, administrative staff and IT infrastructure.
